# Development of an autodissemination strategy for the deployment of novel control agents targeting the common malaria mosquito, *Anopheles quadrimaculatus* say (Diptera: Culicidae)

**DOI:** 10.1371/journal.pntd.0006259

**Published:** 2018-04-11

**Authors:** Daniel R. Swale, Zhilin Li, Jake Z. Kraft, Kristen Healy, Mei Liu, Connie M. David, Zhijun Liu, Lane D. Foil

**Affiliations:** 1 Louisiana State University AgCenter, Department of Entomology, Baton Rouge, LA, United States of America; 2 Louisiana State University AgCenter, School of Renewable Natural Resources, Baton Rouge, LA, United States of America; 3 Louisiana State University, Department of Chemistry, Baton Rouge, LA, United States of America; Centers for Disease Control and Prevention, Puerto Rico, UNITED STATES

## Abstract

**Background:**

The reduced efficacy of current *Anopheline* mosquito control methods underscores the need to develop new methods of control that exploit unique target sites and/or utilizes novel deployment methods. Autodissemination methodologies using insect growth regulators (IGRs) is growing in interest and has been shown to be effective at controlling *Aedes* mosquitoes in semi-field and field environments, yet little information exists for *Anopheline* mosquitoes. Therefore, we tested the hypothesis that female-driven autodissemination of an IGR combined with a new mechanism of action insecticide (Kir channel inhibitor) could be employed to reduce *Anopheline* populations.

**Methodology:**

We studied the ability of three IGRs to be transferred to the larval habitat during oviposition in laboratory and semi-field environments. Adult mosquitoes were exposed to the chemicals for 4 hours immediately after blood feeding and efficacy was tested using classical methodologies, including adult emergence inhibition and High Performance Liquid Chromatography (HPLC). A complete autodissemination design was tested in a semi-field environment.

**Principal findings:**

Larval survivability and adult emergence were significantly reduced in habitats that were visited by novaluron treated adults, but no statistical differences were observed with pyriproxyfen or triflumuron. These data suggested novaluron, but not pyriproxyfen or triflumuron, was horizontally transferred from the adult mosquito to the larval habitat during oviposition. HPLC studies supported the toxicity data and showed that novaluron was present in the majority of larval habitats, suggesting that novaluron can be horizontally transferred by *Anopheles quadrimaculatus*. Importantly, the combination of novaluron and the Kir channel inhibitor, VU041, was capable of reducing adult and larval populations in semi-field environments.

**Conclusions:**

Novaluron can be transferred to the adult at a greater efficacy and/or is not degraded as quickly during the gonotropic cycle when compared to pyriproxyfen or triflumuron. Pending field confirmation, autodissemination approaches with novaluron may be a suitable tool to manage *Anopheles* populations.

## Author summary

Efforts to control the mosquito vector of malaria, *Anopheles gambiae*, have been dominated by the use of insecticide-treated bednets or residual spraying efforts for the previous 2–3 decades. The persistent use of these methods has led to a decline in control efficacy and has highlighted the need to 1) identify novel molecular targets and 2) novel translational deployment methods to control mosquito vectors. To address this, we employed biological and chemical methods to test the hypothesis that insect growth regulators (IGR) are capable of being transferred to an oviposition site at lethal concentrations when *Anopheles* adults are exposed immediately after blood feeding. Subsequently, we tested the hypothesis that K^+^ channel modulators and an IGR used in combination will reduce the mosquito population in a semi-field environment through adult toxicity and IGR transfer. The data presented in this study provides a proof-of-concept that autodissemination methods using specific IGRs and K^+^ channel modulators are potentially capable of reducing the burden of malaria through a method that is novel, cost efficient, long lasting, and requires minimal human intervention.

## Introduction

Mosquitoes are vectors of numerous pathogens that induce extreme morbidity and mortality worldwide. Collectively, the pathogens transmitted by mosquitoes are responsible for the death of hundreds of thousands of people annually and induce debilitating diseases in hundreds of millions more individuals worldwide [1,2]. Control of mosquito-borne diseases has been largely successful by controlling the adult population through the use of synthetic insecticides, such as pyrethroids and organophosphates. While the current methods of vector control have been outstanding, the efficacy of these methods are beginning to decline due to the evolution of biochemical and behavioral resistance to the approved insecticidal classes [3–7]. Considering the threats caused by worldwide insecticide resistance [5,6,8] and the growing threat of ‘secondary’ mosquito vectors (e.g. *Anopheles albimanus*, *An*. *pharoensis)* maintaining the malaria transmission rates [9,10], we are in need of additional and complementary tools for resistance management of mosquito populations. Potential novel methods to reduce populations of both susceptible and resistant mosquitoes include the use of attractive toxic sugar baits, genetic manipulations, paratransgenesis, attractants for trapping, and novel “attract and kill” strategies [11–15].

Autodissemination methods are a type of “attract and kill” system that have been studied over the last decade for use in mosquito control programs targeting container breeding mosquitoes, such as *Aedes aegypti* and *Aedes albopictus* [15,16]. With this approach, female mosquitoes transfer lethal concentrations of an insect growth regulator (IGR) to the larval habitat during oviposition, resulting in a mosquito population reduction through larval control. The autodissemination technique has been shown to induce toxicity to *Aedes aegypti* and *Aedes albopictus* in the laboratory. In semi-field studies, the juvenile hormone analog, pyriproxyfen (PPF), was capable of being transferred to larval habitats during oviposition and reduced emergence rates by 42–100% [16–21]. However, the biology of *Aedes* mosquitoes contributes to the success of autodissemination methods since they oviposit in multiple containers during a single gonotrophic cycle, and share oviposition sites with multiple females [22]. These biological traits enable gravid mosquitoes to be contaminated with PPF immediately prior to oviposition, which reduces the potential for chemical loss during the gonotropic cycle, maximizes the number of IGR transfer events, and amplifies the efficacy of the IGR for reducing emergence.

While there is growing interest in autodissemination techniques in *Anopheline* mosquito control, their biology poses several challenges. *Anopheline* mosquitoes oviposit in aquatic habitats of varying size and stability rather than in containers [23]. *Anopheles gambiae* females have been shown to oviposit in habitats as small and temporary as hippopotami footprints or as large and permanent as Lake Victoria [24]. Therefore, an additional component to the traditional autodissemination technique must be employed to successfully control *Anopheles* using this strategy. A potential alternative for *Anopheles* mosquitoes could be using oviposition attractants to lure *Anophelines* to a specific oviposition site that would facilitate autodissemination. Cedrol, a natural product extracted from cedarwood oil, has been shown to be attractive to gravid *Anopheles gambiae* mosquitoes [25]. Therefore, this product could provide a critical component to an autodissemination system targeting *Anophelines*.

Relatively few studies have explored the potential of using autodissemination methods for controlling *Anopheline* mosquitoes and all of the previous studies have characterized the impact that PPF has in the horizontal transfer system [15,26]. In laboratory studies, Mbare and colleagues demonstrated that gravid *Anopheles gambiae* females can transfer lethal concentrations of PPF to oviposition sites, but noted that PPF exposure must occur within 24 hours of oviposition so that sufficient PPF can be delivered to aquatic habitats [15]. Employing this methodology in the field is a major limitation since adult exposure to the IGR within 24 hours of oviposition is unlikely and this difficulty drove the authors to conclude that autodissemination of pyriproxyfen is not a feasible strategy for *Anopheles gambiae* control [27]. On the contrary, data collected in a semi-field setting targeting a secondary malaria vector, *Anopheles arabiensis*, suggests that PPF is capable of being transferred from resting sites (i.e. clay pots) to the larval habitat during oviposition [26]. These studies raise the intriguing possibility that modification of the autodissemination methods and the inclusion of unexplored IGR’s may produce a method capable of controlling *Anopheles* populations.

In addition to these advancements for developing the horizontal transfer system, novel synthetic insecticides have been recently developed that target inward rectifying potassium (Kir) channels, a superfamily of potassium ion channels that has been shown to play key roles in insects. These compounds are believed to induce their lethality through inhibition of Malpighian tubule function, which is the tissue responsible for processing the blood meal and osmoregulation after consumption of ion-rich blood. The small-molecule, termed VU041, is capable of reducing urine output, preventing blood meal processing after feeding, and inducing mortality after contact exposure to the chemical [28]. Importantly, this class of chemicals has been shown to kill the carbamate- and pyrethroid-resistant strain of *Anopheles gambiae*, known as Akron strain, which are ubiquitously present in Sub-Saharan Africa and Asia [28]. Taken together, the development of the novel Kir channel adulticides, the commercialization of highly toxic IGRs, and the use of gravid attractant cedrol [25] provide a unique opportunity to develop a novel method of reducing *Anopheline* mosquito populations.

Although autodissemination platforms have significant potential to reduce mosquito borne pathogens, the lack of knowledge pertaining to IGRs other than PPF that could be used in autodissemination systems is limiting, and potentially fatal, to the development of autodissemination as a novel translational deployment method for mosquito control. For instance, the inevitable development of PPF resistance will cause autodissemination methods to become completely ineffective since no other IGR with differing mechanism of toxicity has been characterized. The goal of this study was to address this deficit in knowledge by employing biological and chemical methods to test the ability of three IGRs (pyriproxyfen, triflumuron, novaluron) to be horizontally transferred to an oviposition site when *Anopheles* adults are exposed immediately after blood feeding. These methods will highlight additional mechanisms for exposing *Anopheles* to the IGR since contamination at oviposition sites, as is done with *Aedes*, is likely to be an insurmountable challenge. Subsequently, we tested the hypothesis that Kir channel modulators and an IGR used in combination will reduce the mosquito population in a semi-field environment. The data presented in this study provides a proof-of-concept that autodissemination methods using specific IGRs and Kir channel modulators are a plausible method for control of *Anopheline* mosquitoes.

## Methods

### Compounds, solvents, and reagents

**Fig 1:**
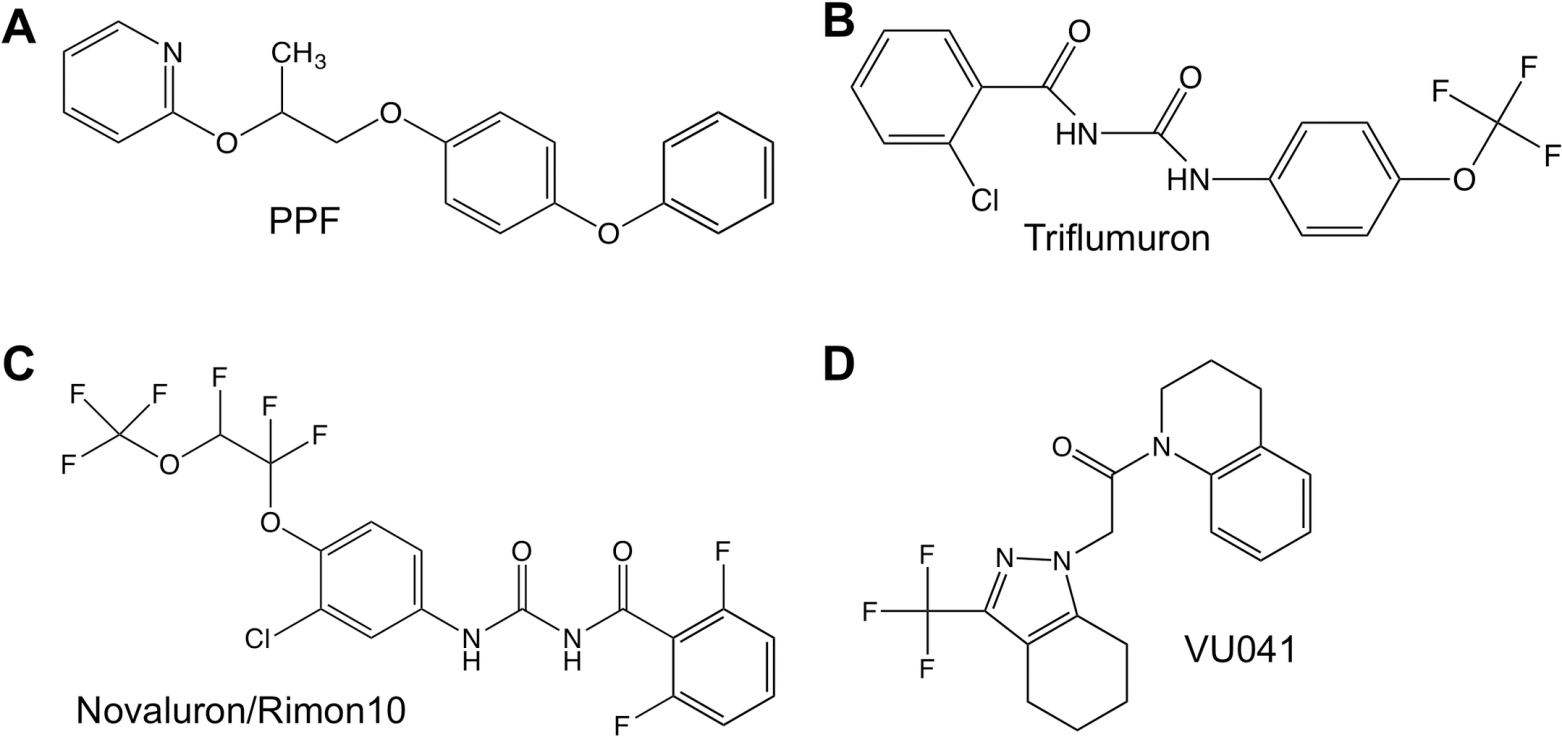
Chemical structures of chemicals used in this study. **A-C)** Chemical structures of insect growth regulators: pyriproxyfen (PPF) or Sumilarv (Sumil.), Triflumuron (Trifl.), and novaluron (noval.) or Rimon EC10 **D)** Chemical structure of the Kir channel inhibitor, VU041.

The Kir channel inhibitor VU041 was originally discovered in a high-throughput screen against the *Anopheles gambiae* Kir1 channel [28] and synthesized in bulk by Molport (Rita, Latvia, Europe). Pyriproxifen (grade: analytical grade) was purchased from Sigma-Aldrich (St. Louis, MO, USA) and formulated product was provided through commercial sources. Technical grade triflumuron was purchased from Santa Cruz Biotechnology Inc. (Dallas, TX, USA). Technical grade novaluron (98%) powder and the formulated product Rimon EC10 was provided by Makhteshim-Agan of North America (Raleigh, NC, USA). Dimethyl sulfoxide (DMSO), acetone, and absolute ethanol were purchased from Sigma-Aldrich. Chemical structures of the insecticides used in this study are shown in Fig 1.

### Mosquitoes

The common malaria mosquito (*Anopheles quadrimaculatus*) was the only species used in this study and adults were provided by the Center for Medical, Agricultural, and Veterinary Entomology (CMAVE), USDA-ARS, Gainesville, FL. The *An*. *quadrimaculatus* colony has been established in the insectary at CMAVE since 1952 and originated from wild caught mosquitoes in Orlando, FL. The mosquitoes were maintained in an environmental chamber set to 27 °C and 75% humidity at a 14:10 light:dark cycle in the Life Sciences Building of Louisiana State University (Baton Rouge, LA, USA) prior to use and provided access *ab libitum* to 10% sucrose.

### Laboratory toxicity assays

IGR toxicity was determined in the laboratory with 3^rd^-instar *An*. *quadrimaculatus* and was measured by the inhibition of adult emergence. IGRs were first dissolved in DMSO or ethanol and then serial dilutions were performed in tap water for each test concentration to result in a final solvent concentration of no greater than 0.01%. Control treatments contained 0.01% solvent only. A total of 25 ultimate instars were placed in a glass dish that contained 25 mL of water, 5 mg of fish food flakes, and total emergence was recorded 72 hours after initiation of the experiment. For the emulsifiable concentrate formulation of novaluron, Rimon EC10, we tested 9 concentrations prepared by serial dilutions in DMSO, following standardized procedures. Each dosage was assayed using the immature first stage larval instar, third stage larval instar, or pupae. A total of 3 replicates were performed per concentration with each replicate consisting of at least 20 individuals for each life stage tested. The % inhibition of emergence was determined based on modifications to those described in Estrada and Mulla [29]. Percent mortality values were obtained for each concentration and were subjected to probit-regression analysis using GraphPad Prism v7.0 (Graphpad Software, LaJolla, CA, USA) to determine the concentration required to inhibit 50% of emergence (IE_50_).

Adult contact toxicity to the Kir channel inhibitor, VU041, was determined against blood fed mosquitoes using a slightly modified W.H.O. protocol [30]. The primary modification was the use of electrostatic netting instead of paper. A range finding assay was performed with 10, 5 and 0.5 mg/mL single treatments after which a subsequent detailed assay followed to determine actual concentration required to kill 50% of the population (LC_50_). A total of 5 concentrations were prepared, and a 15 cm X 10 cm square piece of netting soaked in 2 mL of each concentration of VU041 using 95% ethanol as solvent. Mosquitoes were chilled for 3 min on ice, after which 25 females were placed in the WHO cylindrical holding chamber to acclimatize for one hour. Mosquitoes were then moved to the treatment chamber that contained treated netting and left for 1 hr, after which they were transferred back to the holding chamber and maintained on 10% sugar solution for 24 hrs. Each concentration was repeated in triplicate using different batches of mosquitoes to minimize inter-batch variability, with an ethanol only treated as a vehicle control. Mortality was recorded 24 hr after treatment and experiments having control mortality >20% were discarded. Mortality data were corrected for control mortality and analyzed by log-probit using Poloplus (LeOra Software, Petaluma, CA, USA) or GraphPad Prism v7.0 software to generate the 24 hr LC_50_.

### Exposure of adult female mosquitoes to IGRs

**Fig 2:**
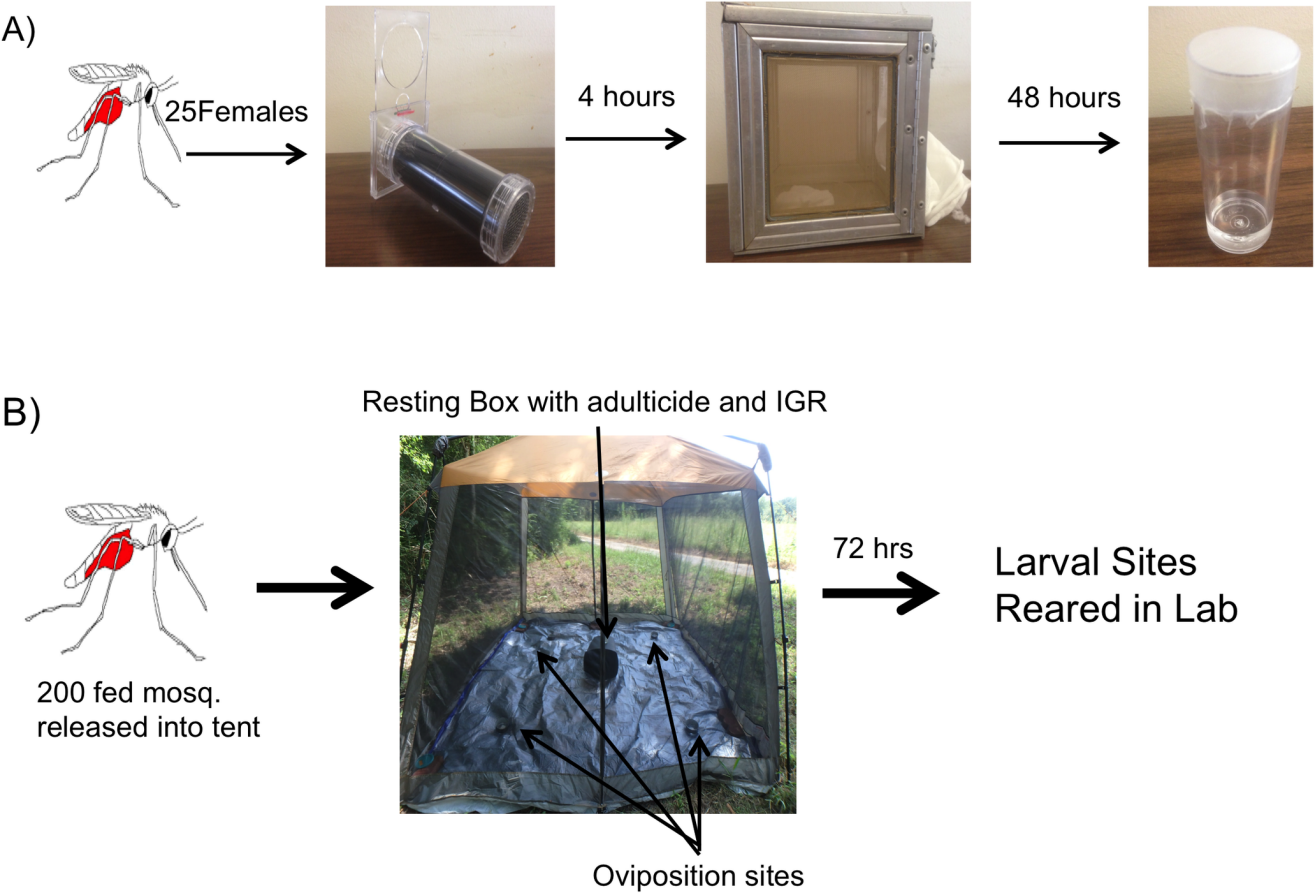
Experimental design for laboratory and semi-field studies. **A)** Schematic diagram showing the methodology used to expose *Anopheles quadrimaculatus* to the IGRs in the laboratory. **B)** Schematic diagram showing the methodology for testing the efficacy of IGRs and adulticides to control the mosquito population in a semi-field environment.

Electrostatic polyester netting purchased from Van Heek Textiles BV (Losser, Netherlands) was used as the material for insecticide delivery. This netting has been shown to deliver an increased mass of insecticide to the mosquito after landing, which is an advantageous property for a horizontal transfer system [31]. The netting was cut into 15 cm X 10 cm squares and treated with the IGRs in two separate manners. Chemicals were dissolved in ethanol and nets were soaked in the IGR solution at a final concentration of approximately 500 μg/cm^2^ for 30 minutes. The nets were dried at room temperature for 1 hour and inserted into one side of a WHO assay tube [30]. Mosquito exposure to IGRs followed the 2006 WHO protocol [30] for contact exposure to insecticides with slight modifications. Briefly, 3–5 day old *An*. *quadrimaculatus* females were blood fed to mimic exposure in the field and 25 fully engorged females were removed immediately after blood feeding and placed in the WHO cylindrical holding chamber to acclimatize for one hour. After acclimatization, mosquitoes were transferred to the treatment chamber that contained netting treated with the chemical. The mosquitoes were removed after 4 hours of exposure in the treatment chamber and transferred to an untreated holding chamber for 48 hours to complete the gonotropic cycle. The mosquitoes were provided access to 10% sucrose solution *ad libitum*. After 48 hours, individual mosquitoes were transferred to vials for analysis of fecundity and IGR transfer (described in section 2.6). A schematic diagram of this method is shown in Fig 2A.

### Determination of egg hatch rates

Two methods were used to determine if the IGR influenced the viability of eggs. First, the blood fed mosquito was exposed to the IGR by methods described in section 2.4 and individual mosquitoes were allowed to oviposit in *Drosophila* tubes containing 1 mL of tap water. Percent hatch rate was determined by comparing total number of eggs at 12 hours post oviposition with the total number of larvae 36 hours post oviposition. Secondly, uncontaminated mosquitoes were allowed to oviposit on tap water that was treated by an IE_50_ of PPF, novaluron, or triflumuron. Percent hatch rate was determined in an identical manner as previously described.

### Transfer of IGRs to larval habitat during oviposition

After the 48-hour holding period to complete the gonotropic cycle, individual mosquitoes were transferred to glass vials with an outer diameter of 28.5 mm. A total of 2 mL of distilled water was added to the vial along with 3 mg of liver powder to serve as a food source for hatched larvae. The total number of eggs per vial was counted immediately after oviposition and the hatch rate was determined for each treatment to determine the viability of the eggs. The efficacy of transfer for the chitin synthesis modulators, novaluron and triflumuron, was determined by the ability of the hatched larvae to molt from the first larval (L_1_) life stage to second larval (L_2_) life stage. However, since PPF is a juvenile hormone analog, adult emergence was the necessary measure of horizontal transfer. Unfortunately, we were not able to measure emergence due to the small volume of water used in each oviposition vial. Therefore, to compare emergence of mosquitoes after oviposition, we used similar methods to those described in Mbare et al [15]. Briefly, a total of ten 4^th^ instar larvae were added to vials that had eggs oviposited and emergence was compared to control vials (vials with eggs from unexposed mosquitoes). A minimum of 25 oviposition vials were used per replicate and a total of 4 replicates were used for novaluron, pyriproxyfen, triflumuron, and control tests. Total replicate numbers were at least 100 mosquitoes and oviposition sites per treatment.

The formulated product of novaluron, Rimon EC10, was used to determine the longevity of the product after a net was treated with the product. Here, we followed the same methodology as described for the technical grade molecules, but soaked the net in a 40 mL of product and placed the netting into an oven at 100°C for 2 hours to reduce the stickiness of the product. Mosquitoes were exposed to the chemical for 4 hours at five separate time points that were separated by 30 days. During the 30 day period, the net was stored in open air at 25 °C and the same net was used in the WHO exposure tube every trial. For replications, a total of 3 separate nets were used and a total of 10 mosquitoes were exposed per time point, per net. Adults that died due to the adhesive nature of the product were discarded from the study. Larval survivability from L_1_ to L_2_ was recorded as previously described.

### High Performance Liquid Chromatography (HPLC) analysis for quantification of IGR presence

HPLC coupled to accurate mass electrospray ionization (ESI) mass spectrometry was utilized in this analysis. The samples were directly injected into mass analyzer and did not go through chromatographic column separation. The injection volume was 1 μL and the compounds were dissolved into acetonitrile. MS conditions were as follows: capillary voltage 3.0 kv, cone voltage 50 v, extractor voltage 2 v, source temperature 120 °C, desolvation temperature 350 °C, desolvation gas (nitrogen) 400 L/h, and cone gas (nitrogen) 50 L/h. Quantitative analysis was performed by monitoring the target ion of the analyte novaluron (m/z 493.7 [M+H]^+^) in selected ion reaction (SIR) monitoring and positive ionization mode. All data were acquired in centroid mode and processed using Mass hunter (Santa Clara, CA, USA).

The HPLC-MS profile for each IGR was determined by injecting a 100 μg/mL and identifying the prominent counts vs. mass-to-charge (m/z) that corresponded to the chemical. Once the m/z for each compound was determined, the limits of detection (LOD) and limits of quantification (LOQ) were identified for novaluron, pyriproxyfen, and triflumuron that would define a threshold of detection of the oviposition sites. Quantification of the IGRs in the oviposition sites were based on a standard curve generated from 5 concentrations (0.1 ppb, 1 ppb, 10 ppb, 25 ppb, and 50 ppb) and is shown in Supplementary Fig 1A. The IGR was considered to be present in the oviposition site if the peak at the expected retention time was within 0.003 units of abundance when compared to the HPLC analysis of the technical grade IGR. The concentration of novaluron in each oviposition site was estimated based on the standard curve using a simple formula of abundance of oviposition site / abundance in known concentration of novaluron. The average concentration shown in Table 1 is the concentration from oviposition sites that were shown to have greater than 15% reduction in survivability.

**Table 1:** HPLC analysis of IGR chemicals in oviposition habitats of *Anopheles* females exposed by contact immediately after blood feeding.

Compound	Number Positive Detected	Mean Concentration (ppb) ± SD
PPF	0/30	< 1
Triflumuron	5/25	<15
Novaluron	13/25	5.2 ± 2.1
Rimon EC10	19/25	27 ± 1.7

The mean concentration for triflumuron, novaluron, and Rimon EC10 was derived out of the samples that exhibited 15% toxicity to the larvae. All PPF samples were tested by HPLC.

### Semi-field experiments

The semi-field experiments described in this study were performed from June 2016 –August 2016 and were performed at LSU AgCenter Botanical Gardens at Burden located in Baton Rouge, LA, USA. The semi-field environment constructed from screened tents with dimensions of 3.03 X 3.03 meters and approximately 2 meters at the highest peak. Flooring was provided by a silver, polyvinyl tarp that was glued to the interior of the tent to prevent mosquito escape. All semi-field environments were located in areas that had significant shade cover.

Each screened tent contained a resting box that was located in the middle of the enclosure. The dimensions of each resting box was 30.5 X 30.5 X 30.5 cm plastic boxes and had a black colored exterior and red colored interior. This MRB design was shown to be highly effective at attracting blood fed *An*. *quadrimaculatus* in Louisiana [32] and was therefore used in this study. The interior of the resting box was lined with netting that was treated with the chemicals in an identical manner as the laboratory assays described in section 2.3. Four separate glass oviposition sites were placed in each corner of the screened tent and each were filled with 100 mL of distilled water (Fig 2B).

Female *Anopheles quadrimaculatus* were blood fed on defibrinated bovine blood using an artificial membrane feeder in the laboratory. All bovine blood was purchased from the vendor Hemostat Laboratories (Dixon, CA, USA; product number DBB1) and all mosquito blood feeding protocols were approved by the LSU AgCenter’s Inter-Institutional Biological and Recombinant DNA Safety Committee (IBRDSC); approval number 02015. Upon completion of blood feeding, 200 freshly blood fed mosquitoes were released into the tent no later than 0800 hours. The mosquitoes were left in the tent for approximately 72 hours to provide enough time for oviposition. After 72 hours the mosquitoes were recaptured using a backpack aspirator and the larval habitats were sealed to prevent evaporation and reared to adult eclosion in growth chambers in the laboratory. Growth chambers were set at 27° C and 70% RH. Methodologies for semi field studies are highlighted in Fig 2B.

### Statistical analysis

**Fig 3:**
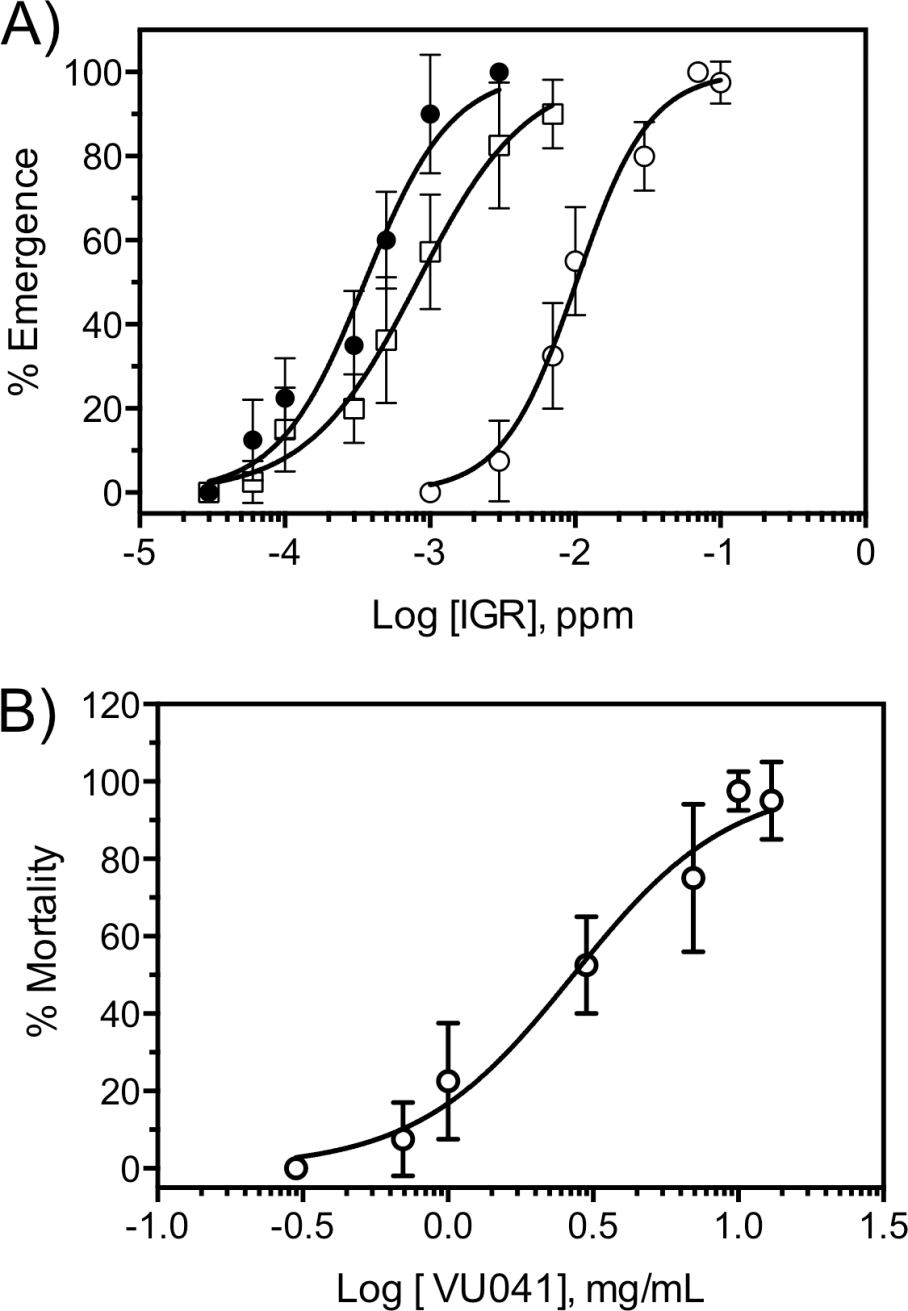
Toxicity of IGRs and VU041 to *An*. *quadrimaculatus*. **A)** Concentration-response curves of triflumuron (closed circles), pyriproxyfen (open squares), and novaluron (open circles) as determined by inhibition of adult emergence after exposure to 4^th^ instars. Each data point represents an average (n = 3) toxicity and each replicate consisted of 30 larvae; totaling 90 mosquitoes. Error bars represent SEM. **B)** Toxicity in blood fed, adult *An*. *quadrimaculatus* 24h after contact exposure to netting treated with VU041. Data points represent a mean mortality (n = 4–8 replicates of 25 mosquitoes tested per dose) and error bars represent SEM.

**Fig 4:**
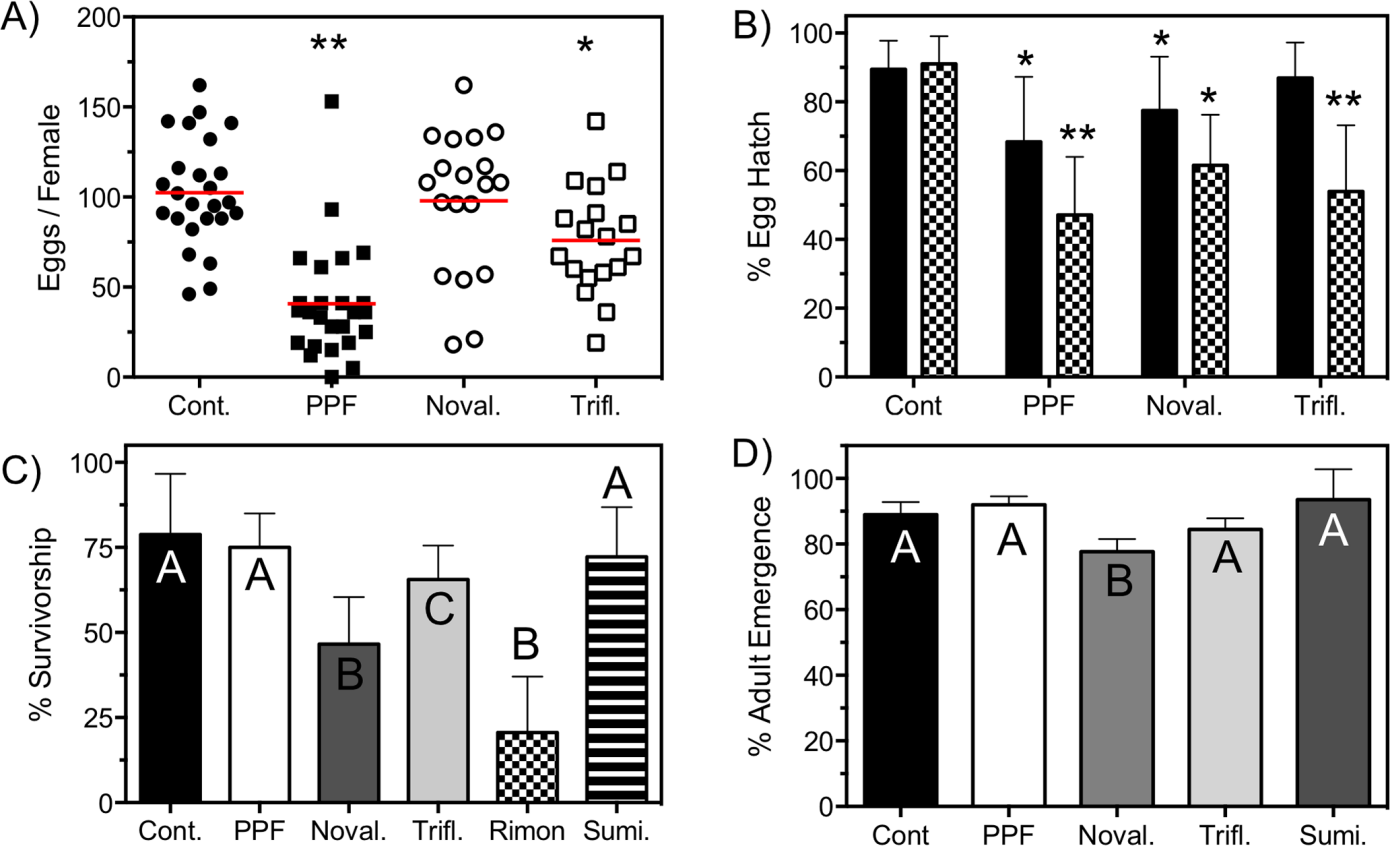
Influence on fecundity and larval survivorship after adult exposure to IGRs. **A)** Total number of eggs laid per female 72 hours after adult contact exposure to IGRs immediately after blood feeding. Control treatment was netting treated with acetone only. Each data point represents the egg output of an individual mosquito. Red bars indicate the median number of eggs laid for each treatment. Asterisks represent statistical significance (P<0.05) when compared to the control (solvent only) treatment. **B)** Percent hatch of the eggs. Solid bars represent the mean % egg hatch in habitats that were contaminated by the adult mosquito during oviposition (horizontal transfer) whereas the checkered bars represent egg hatch of eggs laid in water contaminated with an IGR at the IE_50_ concentration. Bars represent a mean % hatch (n = 25) and error bars represent SEM. Asterisks represent statistical significance (* P<0.05, **P<0.01) when compared to the control treatment for each group, horizontal transfer and IGR treated water. **C)** % survivorship of larvae hatched from a habitat that was oviposited in by adults treated with IGRs. Mortality was measured by counting number of alive 1^st^-instar larvae compared to 2^nd^-instar larvae. Bars represent average survivorship (n = 25) and error bars represent SEM. **D)** % emergence of ultimate instar larvae added into oviposition sites that had eggs laid. Bars represent means (n = 4, 10 mosquitoes per rep) and error bars represent SEM. For all panels, letters indicate statistical categorization of the means as determined by a one-way ANOVA with a multiple comparisons test (P < 0.05). Bars not labeled by the same letter as the control bar represents statistical significance when compared to the control value (P<0.05).

**Fig 5:**
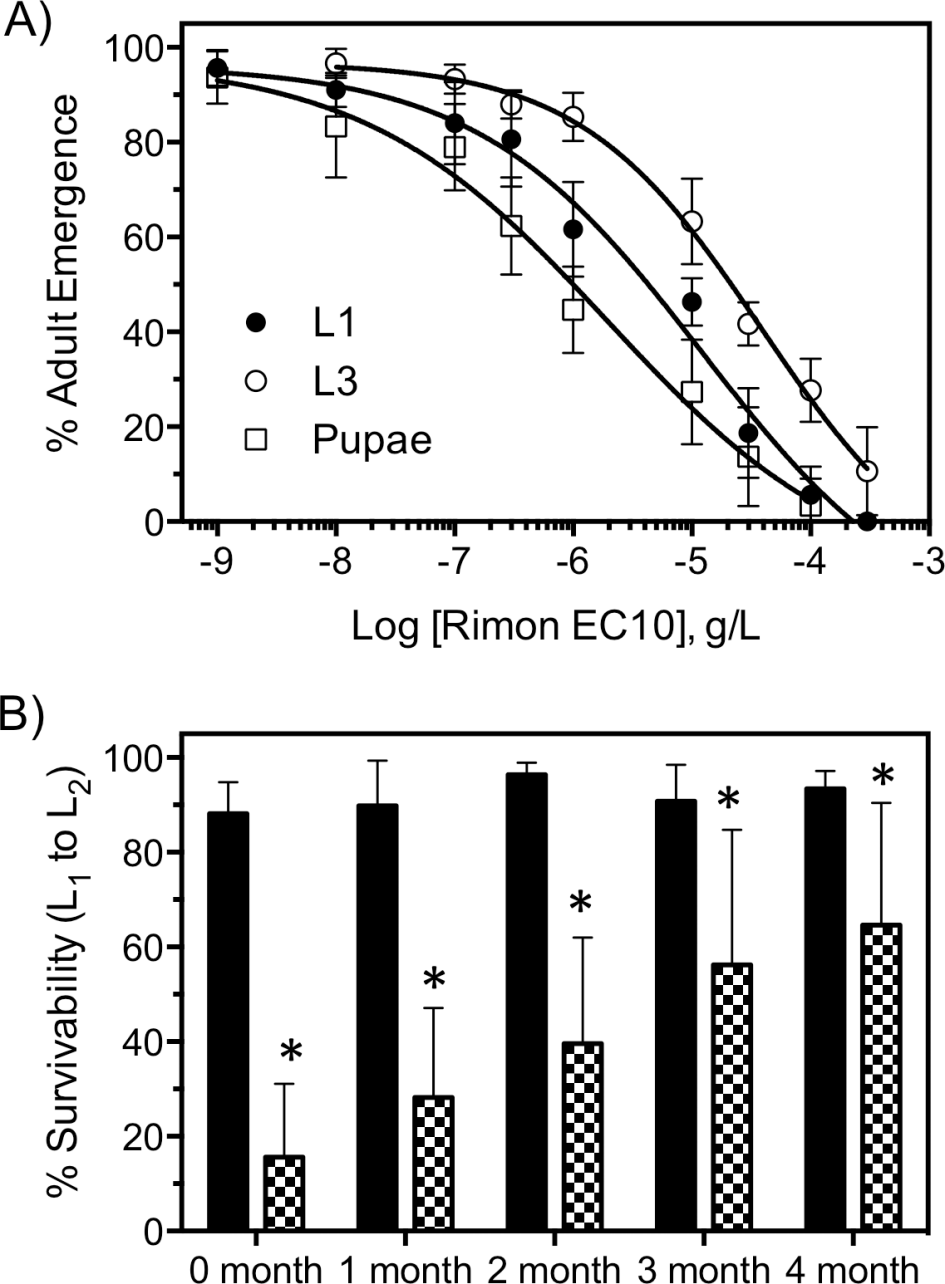
Larval toxicity and time course of horizontal transfer capabilities of Rimon EC10. **A)** Concentration-response curves of Rimon EC10 when exposed to 1^st^-instars (closed circles), 3^rd^-instars (open circles), and pupae (open squares) as determined by inhibition of adult emergence. Each data point represents a mean % emergence derived from 3 replicates and each replicate consisted of 3 replicates of 20 larvae. Error bars represent SEM. **B)** % survivorship of larvae hatched into a habitat that was oviposited in by adults exposed to control (black bars) or Rimon EC10 treated netting (checkered bars) immediately after blood feeding. Mortality was measured by counting number of alive 1^st^-instar larvae compared to 2^nd^-instar larvae. Bars represent average survivorship (n = 25) and error bars represent SEM.

**Fig 6:**
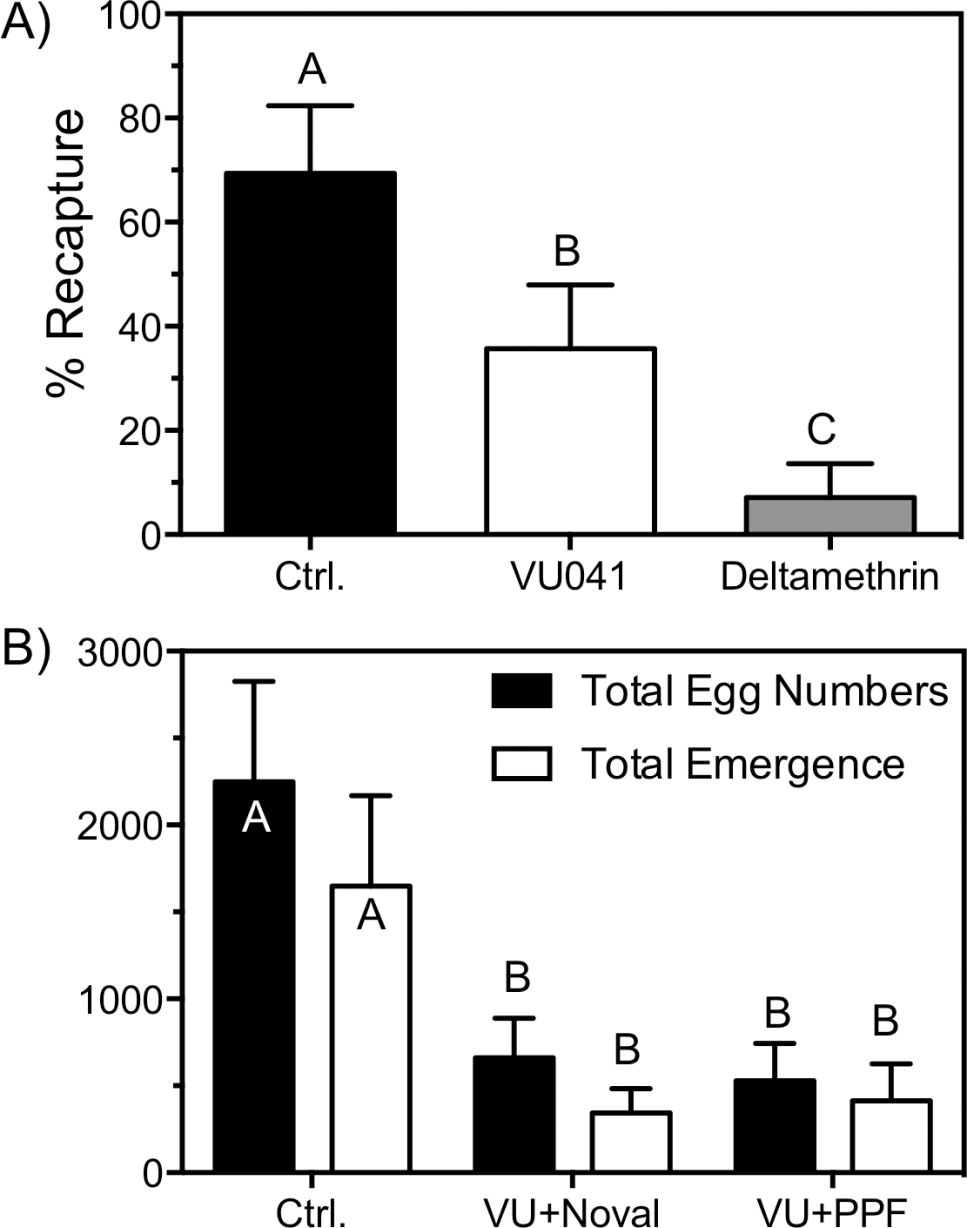
Autodissemination efficacy of novaluron and PPF and toxicity of VU041 in a semi-field environment. **A)** % recapture of adult mosquitoes when exposed to a VU041- or deltamethrin-treated resting boxes. Deltamethrin was tested at an LC99 to determine the visitation rates of mosquitoes to the resting box. Bars represent mean (n = 6) % recapture and error bars represent SEM. **B)** Fecundity and adult emergence of control-, VU041+novaluron-, and VU041+PPF-treated groups. Bars represent mean (n = 15) and error bars represent SEM.

Concentration-response curves (CRC) were used to generate IC_50_ and LC_50_ values using IGRs (Fig 3A) and VU041 (Fig 3B), respectively and were generated by fitting the Hill equation using variable-slope, unconstrained, nonlinear regression analyses performed with GraphPad Prism (GraphPad Software, San Diego, CA). For the CRC’s, each data point represents an average (n = 3) toxicity and each replicate consisted of 30 larvae; totaling 90 mosquitoes. Mean eggs laid per female and % egg hatch (Fig 4A and 4B) were statistically analyzed with the use of an unpaired t-test for each treatment group compared to the mean control values. The % survivorship and adult emergence (Fig 4C and 4D) was analyzed using a one-way ANOVA with a Dunns multiple comparisons post-test whereas the % survivability after Rimon EC10 exposure (Fig 5B) was analyzed by comparing the control survivability with the treated group for each time point using an unpaired *t-*test. For the semi field experiments, the % recapture (Fig 6A) was analyzed by comparing the means of each treatment group to the mean control value with an unpaired *t-*test. The data shown in Fig 6B was analyzed by a one-way ANOVA with a multiple comparisons test to compare each treatment to control for both dependent variables and the mean emergence was compared to the mean egg numbers using a paired *t-*test. Statistical significance for all studies were denoted at P < 0.05.

## Results

### Larval and adult toxicity

Baseline toxicity of the three IGRs used in this study was determined against *An*. *quadrimaculatus* by measuring adult emergence. Adult emergence was determined after 3^rd^-instar *An*. *quadrimaculatus* were treated with pyriproxyfen, triflumuron, and novaluron. Triflumuron was found to be the most toxic IGR with an IE_50_: 0.3 ppb (95% CI: 0.25–0.45 ppm, Hillslope: -1.4, r^2^: 0.92), which was approximately 2.6-fold more toxic than pyriproxyfen (IE_50_: 0.8 ppb, 95% CI: 0.5–1.1 ppm, Hillslope: -1.1, r^2^: 0.92). Novaluron was shown to be the least toxic of the three IGRs studied, yet was still highly toxic with an IE_50_ of 10 ppb (95% CI: 5–15 ppm, Hillslope: -1.5, r^2^: 0.95). These toxicity values are similar to those described in previous studies for other mosquito species (Fig 3A; [33–35].

VU041 was developed to target *Anopheles gambiae* and is toxic after topical exposure (LD_50_ 1.8 μg/mg of mosquito) to non-blood fed mosquitoes, making it the first Kir directed insecticide to exhibit topical activity [28,36,37]. However, the contact toxicity of VU041 was undetermined and is critical for successful inclusion into an autodissemination system. Since resting boxes target freshly blood fed mosquitoes, we evaluated the contact toxicity of VU041 against blood fed female *An*. *quadrimaculatus* using electrostatic netting. VU041 was shown to be toxic after contact exposure with an LC_50_ of 21 μg/cm^2^ (95% CI: 12–34 μg/cm^2^, Hillslope: -1.3, r^2^: 0.93), raising the possibility of using VU041 as one component of an autodissemination platform that would prevent blood meal processing, reduce fecundity, and induce toxicity.

### Influence of IGR exposure to fecundity and egg hatch

Chitin synthesis and juvenile hormone are two critical components to the life cycle of holometabolous insects and studies have previously shown that chitin synthesis modulators are capable of reducing the fecundity of arthropods after exposure [15,38,39], which underlines one of the interests for its inclusion in autodissemination stations. Considering this, we tested the ability of PPF, triflumuron, and novaluron to alter the reproductive capacity of *An*. *quadrimaculatus* from contact exposure immediately after blood feeding. Fig 4A shows the influence of IGR exposure to fecundity as measured through total egg numbers. Control (solvent only) treated animals were found to lay an average of 102.5 ± 29.9 eggs per female and, similar to those described in the literature for other arthropod species. Pyriproxyfen and triflumuron reduced the fecundity by 2.5- and 1.4-fold, respectively when compared to control animals, a statistically significant (P<0.001 and P<0.05, respectively) reduction. Mosquitoes exposed to novaluron after blood feeding did not alter fecundity when compared to untreated mosquitoes with the novaluron-treated animals laying an average of 108 eggs per female.

Others have shown that IGRs are capable of reducing egg viability through the inhibition of oogenesis or hindering egg development after oviposition. We used two distinct methods to determine the influence the IGRs have to the viability of the eggs as a means to identify the optimal compound for use in an autodissemination system targeting *Anopheline* mosquitoes. First, freshly blood fed mosquitoes were exposed to the IGR through contact exposure and the hatch rate was determined based on the number of eggs laid per individual. On average, 91% of the control-treated mosquitoes hatched whereas only 69% and 78% of the eggs hatched in the PPF- and novaluron-treated groups, a statistically significant (P<0.05) reduction. The hatch rate of eggs laid by triflumuron-treated adults was not statistically significant (P = 0.4) when compared to the control with a total of 89.5% of the eggs hatched (Fig 4B, solid bars). The second method used to determine the influence of IGRs on egg viability was to treat the larval habitat with an IGR at IE_50_ prior to oviposition and not expose the adults to any chemical. Treatment of the larval habitat prior to oviposition significantly reduced the viability of the eggs for all of the IGRs studied. PPF and triflumuron caused a 1.8- and 1.7-fold reduction in the hatch, respectively, when compared to control-treated mosquitoes, a statistically significant reduction (P <0.01). Novaluron was found to significantly (P<0.05) reduce egg viability by 1.3-fold when eggs were laid in novaluron-treated water (Fig 4B, checkered bars).

### Toxicity of IGRs after horizontal transfer during oviposition

PPF has been documented to transfer to an oviposition site during oviposition when the adults were exposed within 24 hours to oviposition in *Aedes* mosquitoes. However, since it is not possible to expose *Anophelines* to IGRs immediately prior to oviposition, we tested the hypothesis that IGRs could be transferred to the larval habitat when the adult was exposed to the IGR immediately after blood feeding, which is approximately 72 hours prior to oviposition. To test this hypothesis, we performed two distinct biological assays that quantified the survivability of the larvae from the L_1_ to L_2_ life stage (Fig 4C). An average of 79% ± 18% transitioned from the L_1_ lifestage to the L_2_ lifestage in the control-treated animals. We observed a 72% ± 9% and 71% ± 14% survivability of the PPF- and Sumilarv-treated females, which was not significantly different (P>0.05) when compared to control. This was expected since PPF is a JH mimic and therefore, will not induce a lethal phenotype until adult eclosion. However, a significant reduction in larval survivability was observed when adults were treated with novaluron or triflumuron with only 47% ± 7% (P<0.01) and 66 ± 11% (P<0.05) surviving the L_1_ to L_2_ molt, respectively. Importantly, Rimon EC10 was also shown to transfer to the larval habitat with 20% ± 15% surviving the L_1_ to L_2_ molt, which was statistically significant from the control but not from technical novaluron.

Due to the inability of PPF or Sumilarv to be studied through the methods used with novaluron or triflumuron (Fig 4C), we modified the assay to introduce ultimate instar larvae into oviposition sites that had eggs laid by contaminated adults and quantify adult eclosion. An average of 92% ± 12% of the introduced larvae emerged into adults in the control treatments, which was not significantly different from the PPF or Sumilarv treatment of 92% ± 11 and 91% ± 9%, respectively. Triflumuron treatments yielded an 84% ± 13% emergence, which was a 1.1-fold reduction but not statistically significant (Fig 4D). However, the larvae that were introduced into oviposition sites used by adults exposed to novaluron showed a 1.15-fold reduction in adult emergence, a small but significant (P: 0.045) decrease (Fig 4D). These data mirror those shown in Fig 4C and support the hypothesis that at least novaluron and likely triflumuron are transferred to the larval habitat during oviposition.

### Larval and adult toxicity of formulated novaluron (Rimon10)

Proper formulation of insecticides is oftentimes capable of enhancing toxicity and prolonging efficacy when compared to technical grade insecticides. Therefore, we aimed to determine the larval toxicity and horizontal transfer capability of Rimon EC10, which is the formulated product of novaluron. Rimon EC10 was shown to be highly toxic to *An*. *quadrimaculatus* larvae with pupae being the most sensitive (IE_50_: 1.9 μg/L, 95% CI: 0.8–5.3, Hillslope: 0.61, r^2^: 0.94) when compared to 1^st^ and 2^nd^ instars. When exposed as 1^st^- and 3^rd^-instars, the toxicity was reduced by 6.3- and 20-fold fold or IE_50_ values of 12 μg/L and 38 μg/L, respectively (Fig 5A).

Adult mosquitoes exposed to novaluron were capable of contaminating larval habitats during oviposition (Fig 4C and 4D) and therefore, we aimed to test the efficacy of Rimon EC10 to be transferred during oviposition and the longevity of the nets treated with the product. Fig 5B shows that indeed, Rimon EC10 is capable of being transferred to the larval habitat when the adults are exposed for 1 hour immediately after blood feeding. At time 0, larval survivability from the L1 to L2 life stage was 81% ± 7% whereas the larval survivability in oviposition sites from mosquitoes exposed to Rimon EC10 was 20% ± 15%, a statistically significant (P<0.001) reduction in survivability. Importantly, the ability of Rimon EC10 to be transferred and significantly reduce the larval survivability was maintained as the nets were aged 1-, 2-, 3-, and 4- months with mean larval survivability of 28 ± 15%, 39 ± 22%, 56 ± 28%, and 64 ± 25%, respectively. At the age of 4 months, the efficacy of Rimon EC10 treated nets was reduced by 3.2-fold but still maintained a statistically significant (P<0.05) reduction in survivability when compared to the control (Fig 5B). These data highlight the likelihood of prolonged control when using a formulated product in an autodissemination system and supports future work aimed at enhancing the formulation to be amendable to autodissemination platforms.

### Detection and quantification of IGRs in the larval habitat through HPLC

The biological assays described in section 3.3 and shown in Fig 4 provide convincing evidence that novaluron is transferred to the larval habitat during oviposition, with approximately a 40% and 75% reduction in survivability induced by novaluron and Rimon, respectively. However, we aimed to negate the inherent variability that is present in biological assays by using chemical methods to detect the presence of the IGR. The IGRs were found to have a limit of detection (LOD) of 0.5 ppb, 15 ppb, and 0.5 ppb for novaluron, triflumuron, and pyriproxyfen, respectfully. The limit of quantification (LOQ) was found to be 1 ppb, 20 ppb, and 1 ppb for novaluron, triflumuron, and pyriproxyfen, respectively.

According to the larval toxicity curve shown in Fig 3A, an approximate concentration of 7 ppb and 20 ppm would be analogous to an LC_40_ and LC_75_, respectively. HPLC studies were performed on the sites that displayed mortality and data indicate that novaluron was transferred in 13 out of 25 oviposition sites at an estimated mean concentration of 5.2 ± 2.1 ppb (Table 1). This mean concentration is within the range to yield a larval mortality of approximately 40%, which is what was observed in Fig 4C. Similarly, Rimon EC10 was detected in 19 out of 25 oviposition sites and was detected at a mean concentration of 27 ± 1.7 ppb, which is above the concentration expected to yield 75% larval mortality (Table 1). It is important to note that the mosquitoes exposed to Rimon EC10 and, to a lesser extent technical grade novaluron, were observed to sit on the water surface for longer durations and more frequently than those exposed to PPF or triflumuron, which is potentially the root cause for increased chemical transfer. Representative HPLC plots for novaluron and Rimon are shown in S1B and S1C Fig, respectively. PPF was not detected in any of the oviposition sites (n = 25) and therefore, if any PPF was transferred then it was below the LOD (0.5 ppb) (Table 1). Triflumuron was detected in 5 out of 25 oviposition sites, but all samples were below the LOQ and could not be accurately quantified. For the semi-field oviposition sites, novaluron was detected in 3/10 and 4/12 sites studied for novaluron and RimonEC10 treatment groups but all positive samples were below the limits of quantification. No traces of PPF or triflumuron were detected in any of the studied oviposition sites.

### Efficacy of autodissemination with *An*. *quadrimaculatus* in semi-field conditions

Although data collected in the laboratory suggest novaluron can be transferred to the larval habitat, it is essential to determine the efficacy of VU041 and novaluron in a semi-field environment to determine the potential utility of the compounds for eventual deployment in a vector control program. Therefore, Fig 6 (solid bars) highlights the efficacy of VU041 to reduce the population of blood fed *An*. *quadrimaculatus* in a semi field environment when electrostatic nets were treated and hung in a resting box. An average of 69% of mosquitoes in the control treatments were recaptured 72 hours after introduction into the semi-field environment whereas only 38% were recaptured in the VU041 (LC_70_) groups, a statistically significant reduction (P<0.05) but less than the expected mortality (Fig 6A). To determine if the reduced toxicity in the semi-field setting was due to reduced visitation to the resting box, we treated a net lined resting box with an LC_99_ (200 μg/cm^2^) of deltamethrin and observed a 90% reduction of recapture when compared to control. These data suggest that 1) approximately 90% of the blood fed mosquitoes are visiting the resting box and 2) VU041 is capable of reducing the adult population in a semi-field environment after contact exposure but the efficacy is reduced in the field when compared to the laboratory. However, acute toxicity is not absolutely necessary to reduce the population of mosquitoes due to the ability of VU041 to reduce fecundity through inhibition of blood meal processing [28].

To test the hypothesis that reduced fecundity by VU041 exposure is capable of reducing populations, we compared fecundity of control- and VU041- (LC_70_) exposed mosquitoes in a semi field setting (Fig 6B, solid bars). Although acute toxicity was reduced (Fig 6A), a 70–75% reduction in fecundity was observed in VU041-treated groups compared with the control suggesting that VU041 is capable of reducing the mosquito population through reduced fecundity. Furthermore, a combination of novaluron and VU041 in the resting box yielded an adult emergence rate (total eggs/total emergence) of 52 ± 7% whereas control treated mosquitoes had an adult emergence rate of 73 ± 12%, a relevant but not statistically significant reduction (P: 0.06) in adult eclosion (Fig 6B). Importantly, the adult emergence rate of pyriproxyfen-treated animals was found to be 78 ± 4%, which is not statistically significant when compared to control treatments (Fig 6B).

## Discussion

The interest in autodissemination methods for mosquito control has grown significantly throughout the past decade due to the high toxicity of IGRs, the relatively low cost of IGRs, limited off-target toxicity, and the minimal human intervention required for successful implementation. The greatest control of field populations was obtained when luring gravid females to PPF-contaminated bait stations immediately prior to oviposition, which is possible in *Aedes* since they oviposit in container habitats. However, autodissemination of PPF with bait stations is impractical due to the differing oviposition biology [27]. Studies have shown that, although PPF was capable of being transferred during *Anopheles* oviposition, the adult mosquito must be contaminated with PPF within 24 hours prior to oviposition in order to obtain reduction of adult emergence [15,26]. Considering this, we aimed to test the hypothesis that chitin synthesis inhibitors are capable of transferring lethal concentrations to the larval habitat during oviposition when the female was exposed immediately after blood feeding.

The data presented in this manuscript provide clear evidence that autodissemination methods represent a potentially viable approach for controlling the population levels of *Anopheles quadrimaculatus*, and likely other *Anopheles* species, when adult mosquitoes are exposed to the IGR immediately after blood feeding. This is the first report suggesting the autodissemination approach could be employed for controlling *Anopheline* mosquitoes with the data directly pointing to *An*. *quadrimaculatus* but potentially expanding to other species *Anopheles*. Here, we used biological and chemical methodologies to show that novaluron can be transferred to a larval habitat at concentrations lethal to *Anopheles quadrimaculatus* mosquitoes, as a 22% reduction in adult emergence was observed in the laboratory and semi-field studies (Fig 6B). These data were supported through HPLC data that showed the concentration of novaluron to be what was expected based on the larval toxicity curve (Fig 3A) and toxicity from horizontal transfer (Fig 4C). On the contrary, no significant differences of adult emergence were observed in oviposition sites visited by PPF exposed adults, which was supported through no detection in HPLC studies.

The finding that novaluron is transferred to the oviposition site while PPF is not transferred is surprising considering the similar physiochemical properties of the two compounds. Novaluron persistence is slightly more favorable than PPF with t_1/2_ hydrolysis of 101 days and a t_1/2 photolysis_ of 139 days, whereas PPF has a t_1/2_ hydrolysis of 200 days and a t_1/2_ photolysis of only 16 days, which suggests novaluron is a better candidate for prolonged field uses. Also, novaluron and PPF are highly lipophilic with an octanol/water partition coefficient (LogP) of 5.27 and 5.37, respectively [40,41], which suggests that it is capable of transferring and remaining on the lipid rich cuticle of the female mosquito after landing on the contaminated netting. Considering the similar chemical profile and toxicity of the two chemicals, one potential factor that leads to differing transfer capabilities is the chemical interactions with the electrostatic netting that enhances mosquito contamination with novaluron, but not PPF. Secondly, PPF has been shown to be capable of being transferred at concentrations that induce toxicity when the mosquito is exposed within 24 hours of oviposition [15,27]. Similarly, HPLC analysis has revealed that early exposure to PPF resulted in significant loss of PPF before oviposition [21]. This timeframe of exposure is approximately 24–36 hours after mosquitoes were exposed in the current study, indicating that PPF was likely removed from the cuticle during the gonotropic cycle. Although HPLC data was not performed on the mosquitoes during the gonotropic cycle, the biological data combined with the HPLC analysis of oviposition sites confirms that novaluron remains on the insect cuticle throughout the gonotropic cycle and suggests it is possible to contaminate mosquitoes through novaluron-treated resting sites that are visited immediately after blood feeding [42]. Further analysis of this claim is required to further validate the horizontal transfer properties of novaluron.

Since the laboratory studies shown in Fig 4 suggest that it is possible for mosquitoes to be exposed to novaluron immediately after blood feeding and still achieve horizontal transfer, we tested the hypothesis that larval populations would be reduced when exposed through a novaluron-treated resting box in a semi-field environment. Indeed, the semi-field experiments support those collected in the laboratory and show a numerical reduction of adult emergence when mosquitoes were provided a novaluron-treated resting box in the semi-field environment when compared to control or PPF-treated resting boxes. Although increased larval mortality was observed in novaluron treatment groups through semi-field studies, autodissemination of IGRs for *Anopheles* control is significantly more challenging due to diverse oviposition habitats. The varying volumes of water within the oviposition habitats in the field alter the concentration of the IGR that is transferred between habitats and intuitively yields dramatically different levels of larval toxicity per habitat. This limits translation of data from the semi-field to field habitats. However, the mechanism of toxicity of novaluron raises the potential to mitigate some problems stemming from different oviposition habitat sizes. Briefly, novaluron targets L_1_ whereas PPF targets L_5_ lifestages and therefore novaluron requires less active ingredient to induce lethality. Secondly, novaluron does not need to remain in the habitat throughout larval development to induce toxicity due to targeting L_1_, and lastly, the distribution of novaluron in the water column is less important since L_1_ mosquitoes spend the majority of their time near the water surface. Subsequent studies are needed to validate this hypothesis and further test the applicability of novaluron to be added to autodissemination platforms for mosquito control.

To further enhance the autodissemination platform, we tested the hypothesis that inclusion of an adulticide into IGR-treated resting boxes would reduce the adult population prior to oviposition and would represent an avenue to reduce the onset of insecticide resistance to either the adulticide or the IGR due to the chemicals exploiting two distinct mechanisms of action against two distinct life stages. The Kir channel inhibitor, VU041, was an ideal candidate since it is toxic through contact exposure (Fig 3B) and also capable of reducing blood meal processing and fecundity at a sub-lethal concentrations [28]. Further, VU041 has been shown to be lethal to the carbamate and pyrethroid resistant strain of *Anopheles gambiae* that is currently widespread in malaria endemic regions and is a critical consideration during the development of platforms designed for potential use in Sub-Saharan Africa. Indeed, VU041 treated resting boxes reduced the percent recaptured adults and total number of eggs, indicating VU041 mediated adult toxicity and reduced fecundity, respectively. The resting boxes were treated with an LC_70_ of VU041 to represent a population that has developed slight resistance to VU041, which enabled the analysis of the horizontally transferred IGR to control the “resistant” population. Maintaining the trend, mosquitoes exposed to novaluron-treated resting boxes, but not PPF-treated boxes, were capable of reducing the emergence rate of the eggs that were subsequently oviposited, suggesting that a combination of an adulticide-larvicide is a viable control strategy.

Although acute toxicity is the primary method for reducing the mosquito population in the current control programs, there are additional methods that can reduce the mosquito population without the use of lethal chemicals. For instance, reducing fecundity and/or preventing egg hatch is a viable method for population reduction and some IGRs have been attributed to reducing the reproductive fitness of female arthropods [15,38,39,43–45]. Specifically, pyriproxyfen has been shown to induce sterility to female mosquitoes and, although our data don't support sterility, we did observe a significant reduction in fecundity (Fig 4A). For this reason, it has been considered that integration of PPF into currently deployed insecticides would aid in the resistance management to pyrethroids by acting as a mechanism to kill pyrethroid resistant offspring [46,47]. However, it is plausible to suggest that reduced fecundity would prevent oviposition and therefore, reduce the horizontal transfer of PPF and fail to control resistant offspring. Though a different mechanism of action than PPF, we tested the ability of novaluron and triflumuron to alter the reproductive fitness of *An*. *quadrimaculatus* females. Although L4 mosquitoes exposed to an LC_90_ of novaluron reduced the fecundity of *Culex pipiens [48]*, our data suggest that adults exposed to novaluron does not affect the fecundity of *An*. *quadrimaculatus*, confirming the results of previously published studies with other arthropod species (Fig 4A). However, adult exposure to novaluron reduced the viability of the eggs as was measured through a significantly reduced hatch rate (Fig 4B), which has been previously documented in Colorado Potato Beetles [49]. These data suggest that novaluron may circumvent the problem of reduced oviposition likely in PPF treated mosquitoes since novaluron did not alter fecundity. These data imply that novaluron treated mosquitoes will still oviposit and transfer novaluron to control pyrethroid resistant mosquitoes through reduced hatch rate or novaluron-mediated toxicity. Although not mechanistically defined, the reduced viability of eggs after exposure to novaluron and the related chemical, diflubenzuron, is thought to be through transovarial transfer to eggs [50,51]. Further experimentation is required to determine if use of novaluron can reduce wild mosquito populations through reduced reproductive fitness.

To conclude, autodissemination of PPF has proven effective in *Aedes* control programs and one report has shown success in reducing *Anopheline* populations in semi-field conditions. Yet, sustainable autodissemination methods for control of wild *Anopheles* populations has not been successfully developed or implemented. Further, the inevitable development of PPF resistance will cause autodissemination methods to become completely ineffective since no other IGR with differing mechanism of toxicity has been characterized. We present clear evidence that the chitin synthesis inhibitor, novaluron, is capable of remaining on the adult mosquito for the duration of the gonotropic cycle and contaminating the larval habitat during oviposition at concentrations lethal to L_1_ *An*. *quadrimaculatus*. Importantly, quantification of novaluron in the oviposition sites overlays toxicity data collected through biological assays, which to our knowledge, is the first report to use chemical methodology to quantify the horizontally transferred chemical. The data collected in this study are promising and suggest that novaluron is a potential candidate to be included in autodissemination systems for control of *Anopheles quadrimaculatus*, and likely other *Anopheles* species, through a method that is novel, cost efficient, long lasting (Fig 5B), and requires minimal human intervention. However, prior to use in Sub-Saharan Africa future studies are required for 1) efficacy to the African malaria mosquito, *Anopheles gambiae*, 2) further validation of novaluron transfer during oviposition, 3) determination of number of mosquitoes per volume of oviposition site to maintain lethal concentrations, and 4) stability of the IGRs in field oviposition habitats.

## Supporting information

S1 Fig Representative chromatogram plots for the detection of novaluron. Representative chromatograms for three concentrations of technical novaluron (A), from three oviposition sites that were visited by novaluron exposed mosquitoes (B), and Rimon EC10 exposed mosquitoes (C).
(TIF)
