# Supplementary figures and images for: Development of an autodissemination strategy for the deployment of novel control agents targeting the common malaria mosquito, Anopheles quadrimaculatus say (Diptera: Culicidae)
Source: PLoS Negl Trop Dis. 2018 Apr 11;12(4):e0006259. doi: 10.1371/journal.pntd.0006259 (PMC5894962; doi:10.1371/journal.pntd.0006259)

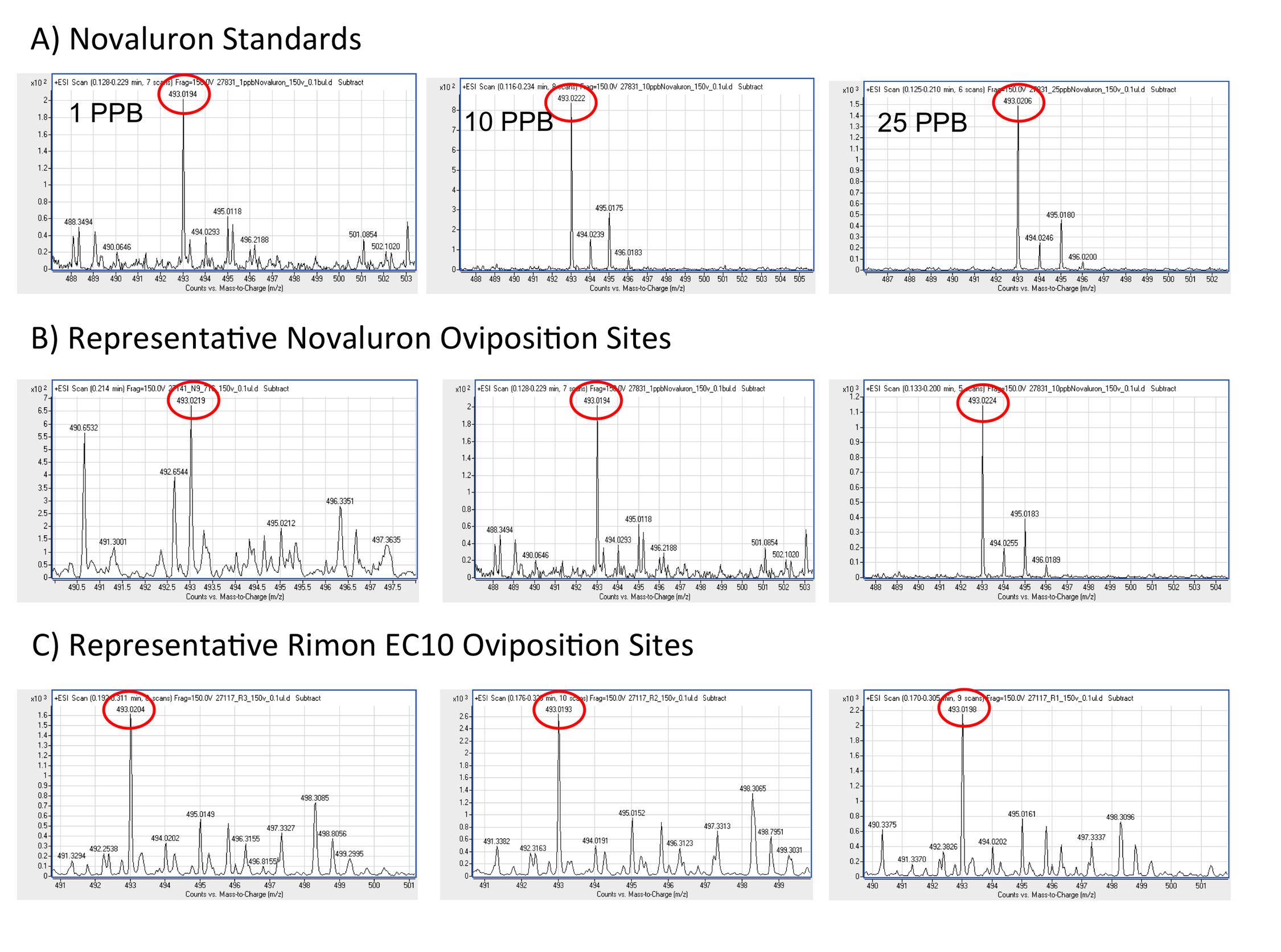

Supplement: S1 Fig — Representative chromatograms for three concentrations of technical novaluron (A), from three oviposition sites that were visited by novaluron exposed mosquitoes (B), and Rimon EC10 exposed mosquitoes (C). (TIF) [file pntd.0006259.s001.tif]
